# Supplementary material for: Transcriptomic Analysis for Diurnal Temperature Differences Reveals Gene-Regulation-Network Response to Accumulation of Bioactive Ingredients of Protocorm-like Bodies in Dendrobium officinale
Source: Plants (Basel). 2024 Mar 18;13(6):874. doi: 10.3390/plants13060874 (PMC10975105; doi:10.3390/plants13060874)
Supplement: Supplementary file 1 [file plants-13-00874-s001.zip › Table S1-7.pdf]

Transcriptomic Analysis for Diurnal Temperature Differences Reveals Gene-Regulation-Network Response to Accumulation of Bioactive Ingredients of Protocorm-Like Bodies in *Dendrobium officinale*

Qingqing Chen, Chunyu Zhang,Xiaoqiong Xu,Yukun Chen,Congqiao Wang, Zhongxiong Lai*

1 Institute of Horticultural Biotechnology, Fujian Agriculture and Forestry University,Fuzhou,Fujian,China; qingqing Chen: qingqing_chen777@163.com; Chunyu Zhang: zcynhba@163.com; Xiaoqiong Xu: XuXQ0921@163.com; Yukun Chen: cyk68@163.com; Congqiao Wang: wcq13313983179@126.com.
*Correspondence: Zhongxiong Lai: Laizx02@.fafu.edu.cn

Table S1 GO enrichment analysis of DEGs

Groups	functional classes	Gene Ontology term	Cluster frequency	Genome frequency of use	Corrected P-value
	MF	DNA photolyase activity	9 out of 1030 genes, 0.9%	9 out of 9095 genes, 0.1%	1.32E-06
	CC	nucleosome	20 out of 794 genes, 2.5%	49 out of 7539 genes, 0.6%	6.64E-06
	CC	DNA bending complex	20 out of 794 genes, 2.5%	49 out of 7539 genes, 0.6%	6.64E-06
	CC	DNA packaging complex	20 out of 794 genes, 2.5%	50 out of 7539 genes, 0.7%	9.99E-06
	CC	chromatin	24 out of 794 genes, 3.0%	73 out of 7539 genes, 1.0%	3.88E-05
	CC	protein-DNA complex	22 out of 794 genes, 2.8%	64 out of 7539 genes, 0.8%	5.19E-05
	MF	chlorophyll binding	19 out of 1030 genes, 1.8%	50 out of 9095 genes, 0.5%	3.90E-04
	CC	photosystem I	12 out of 794 genes, 1.5%	26 out of 7539 genes, 0.3%	8.30E-04
	BP	protein-chromophore linkage	18 out of 860 genes, 2.1%	49 out of 8096 genes, 0.6%	1.23E-03
	CC	photosystem	19 out of 794 genes, 2.4%	65 out of 7539 genes, 0.9%	4.97E-03
Comparison A	CC	plastid thylakoid membrane	36 out of 794 genes, 4.5%	171 out of 7539 genes, 2.3%	6.70E-03
	BP	toxin metabolic process	6 out of 860 genes, 0.7%	7 out of 8096 genes, 0.1%	9.43E-03
	BP	toxin catabolic process	6 out of 860 genes, 0.7%	7 out of 8096 genes, 0.1%	9.43E-03
	BP	secondary metabolite catabolic process	6 out of 860 genes, 0.7%	7 out of 8096 genes, 0.1%	9.43E-03
	CC	plastid	159 out of 794 genes, 20.0%	1140 out of 7539 genes, 15.1%	9.92E-03
	MF	protein heterodimerization activity	19 out of 1030 genes, 1.8%	61 out of 9095 genes, 0.7%	1.14E-02
	CC	thylakoid membrane	37 out of 794 genes, 4.7%	183 out of 7539 genes, 2.4%	1.31E-02
	CC	chloroplast thylakoid membrane	35 out of 794 genes, 4.4%	170 out of 7539 genes, 2.3%	1.40E-02
	CC	thylakoid part	40 out of 794 genes, 5.0%	209 out of 7539 genes, 2.8%	2.40E-02
	CC	photosynthetic membrane	38 out of 794 genes, 4.8%	196 out of 7539 genes, 2.6%	2.67E-02

	MF	DNA photolyase activity	8 out of 1004 genes, 0.8%	9 out of 9095 genes, 0.1%	7.70E-05
	CC	membrane	499 out of 856 genes, 58.3%	3898 out of 7539 genes, 51.7%	4.69E-03
	CC	plastoglobule	10 out of 856 genes, 1.2%	21 out of 7539 genes, 0.3%	7.40E-03
	CC	photosystem I	10 out of 856 genes, 1.2%	26 out of 7539 genes, 0.3%	6.55E-02
	MF	iron ion binding	31 out of 1004 genes, 3.1%	149 out of 9095 genes, 1.6%	1.51E-01
	BP	response to stimulus	151 out of 871 genes, 17.3%	1071 out of 8096 genes, 13.2%	1.51E-01
	BP	iron-sulfur cluster assembly	8 out of 871 genes, 0.9%	17 out of 8096 genes, 0.2%	1.78E-01
	BP	metallo-sulfur cluster assembly	8 out of 871 genes, 0.9%	17 out of 8096 genes, 0.2%	1.78E-01
	MF	carbon-carbon lyase activity	20 out of 1004 genes, 2.0%	83 out of 9095 genes, 0.9%	2.41E-01
	BP	reductive pentose-phosphate cycle	5 out of 871 genes, 0.6%	7 out of 8096 genes, 0.1%	2.55E-01
	BP	photosynthesis, dark reaction	5 out of 871 genes, 0.6%	7 out of 8096 genes, 0.1%	2.55E-01
Comparison B	CC	integral component of membrane	321 out of 856 genes, 37.5%	2480 out of 7539 genes, 32.9%	2.88E-01
	CC	plastid	160 out of 856 genes, 18.7%	1140 out of 7539 genes, 15.1%	2.91E-01
	MF	organic anion transmembrane transporter activity	16 out of 1004 genes, 1.6%	62 out of 9095 genes, 0.7%	3.82E-01
	MF	anion transmembrane transporter activity	24 out of 1004 genes, 2.4%	111 out of 9095 genes, 1.2%	3.86E-01
	CC	intrinsic component of membrane	322 out of 856 genes, 37.6%	2499 out of 7539 genes, 33.1%	3.88E-01
	CC	photosystem	16 out of 856 genes, 1.9%	65 out of 7539 genes, 0.9%	4.01E-01
	CC	intrinsic component of mitochondrial inner membrane	4 out of 856 genes, 0.5%	6 out of 7539 genes, 0.1%	4.12E-01
	CC	integral component of mitochondrial inner membrane	4 out of 856 genes, 0.5%	6 out of 7539 genes, 0.1%	4.12E-01
	BP	response to UV	6 out of 871 genes, 0.7%	11 out of 8096 genes, 0.1%	4.48E-01
	CC	membrane	415 out of 705 genes, 58.9%	3898 out of 7539 genes, 51.7%	5.98E-03
	MF	oxidoreductase activity, acting on paired donors, with incorporation or reduction of molecular oxygen	27 out of 901 genes, 3.0%	122 out of 9095 genes, 1.3%	1.93E-02
Comparison C	BP	secondary metabolic process	13 out of 774 genes, 1.7%	44 out of 8096 genes, 0.5%	1.49E-01
	MF	oxidoreductase activity, acting on paired donors, with incorporation or reduction of molecular oxygen, NAD(P)H as one donor, and incorporation of one atom of oxygen	12 out of 901 genes, 1.3%	41 out of 9095 genes, 0.5%	1.80E-01
	CC	intrinsic component of plasma membrane	22 out of 705 genes, 3.1%	118 out of 7539 genes, 1.6%	2.00E-01

MF	oxidoreductase activity, oxidizing metal ions	6 out of 901 genes, 0.7%	12 out of 9095 genes, 0.1%	2.11E-01
BP	oxylipin metabolic process	5 out of 774 genes, 0.6%	8 out of 8096 genes, 0.1%	3.27E-01
BP	oxylipin biosynthetic process	5 out of 774 genes, 0.6%	8 out of 8096 genes, 0.1%	3.27E-01
BP	single-organism process	452 out of 774 genes, 58.4%	4255 out of 8096 genes, 52.6%	3.27E-01
CC	integral component of plasma membrane	19 out of 705 genes, 2.7%	101 out of 7539 genes, 1.3%	3.77E-01
MF	linoleate 13S-lipoxygenase activity	3 out of 901 genes, 0.3%	3 out of 9095 genes, 0.0%	4.03E-01
MF	trans-cinnamate 4-monooxygenase activity	3 out of 901 genes, 0.3%	3 out of 9095 genes, 0.0%	4.03E-01
MF	anion transmembrane transporter activity	22 out of 901 genes, 2.4%	111 out of 9095 genes, 1.2%	4.71E-01
CC	PCNA complex	3 out of 705 genes, 0.4%	4 out of 7539 genes, 0.1%	5.00E-01
CC	DNA polymerase processivity factor complex	3 out of 705 genes, 0.4%	4 out of 7539 genes, 0.1%	5.00E-01
MF	ammonia-lyase activity	4 out of 901 genes, 0.4%	6 out of 9095 genes, 0.1%	5.07E-01
MF	oxidoreductase activity	145 out of 901 genes, 16.1%	1165 out of 9095 genes, 12.8%	6.05E-01
CC	plasma membrane part	25 out of 705 genes, 3.5%	153 out of 7539 genes, 2.0%	6.44E-01
MF	organic acid transmembrane transporter activity	14 out of 901 genes, 1.6%	60 out of 9095 genes, 0.7%	7.44E-01
BP	negative regulation of cofactor metabolic process	3 out of 774 genes, 0.4%	3 out of 8096 genes, 0.0%	8.24E-01

ComparisonA (25/13 ℃ vs. 13/13 ℃); ComparisonB (13/13℃ vs. 25/25 ℃); ComparisonC (25/13 ℃ vs. 25/25 ℃); DEGs ($\log_2^{Fold(change)} \geqslant 1$ or FDR $\leqslant$ 0.01).

Table S2. The top 20 KEGG pathways enriched by DEGs

	Enriched KEGG Pathway	Qvalue		
		ComparisonA	Comparison B	Comparison C
1	Biosynthesis of secondary metabolites	2.42E-01	4.70E-04	1.11E-04
2	Carotenoid biosynthesis	4.57E-02	2.97E-02	5.92E-02
3	Flavonoid biosynthesis	9.58E-02	3.32E-03	8.69E-06
4	Glyoxylate and dicarboxylate metabolism	2.31E-01	2.33E-01	
5	Sphingolipid metabolism	2.18E-01	1.25E-01	
6	Glycine, serine and threonine metabolism	1.50E-01	1.92E-01	
7	Circadian rhythm-plant	1.41E-01	7.05E-02	
8	Photosynthesis-antenna proteins	1.12E-02	4.70E-04	
9	ABC transporters	1.41E-01		2.31E-02
10	Diterpenoid biosynthesis	9.58E-02		5.91E-02
11	Ribosome biogenesis in eukaryotes	9.54E-02		4.76E-04
12	RNA degradation		1.25E-01	7.26E-02
13	Degradation of aromatic compounds		1.51E-01	6.01E-02
14	Zeatin biosynthesis		1.31E-01	1.63E-02
15	Plant hormone signal transduction		7.05E-02	7.60E-04
16	Stilbenoid, diarylheptanoid and gingerol biosynthesis		5.40E-02	5.19E-04
17	Phenylpropanoid biosynthesis		5.37E-02	2.39E-05
18	Flavone and flavonol biosynthesis		4.94E-03	8.69E-06
19	Lysine degradation	2.31E-01		
20	Photosynthesis	2.18E-01		
21	Nucleotide excision repair	2.17E-01		
22	Glycosphingolipid biosynthesis-globo series	1.74E-01		
23	Carbon fixation in photosynthetic organisms	1.68E-01		
24	Other glycan degradation	1.41E-01		
25	Homologous recombination	9.58E-02		
26	DNA replication	2.97E-02		
27	Mismatch repair	1.97E-03		
28	Limonene and pinene degradation		2.62E-01	
29	Glycerolipid metabolism		2.27E-01	
30	Metabolic pathways		1.39E-01	
31	Biosynthesis of unsaturated fatty acids		1.31E-01	
32	Sesquiterpenoid and triterpenoid biosynthesis		7.05E-02	
33	Basal transcription factors			7.26E-02
34	Pentose phosphate pathway			5.91E-02
35	Plant-pathogen interaction			5.91E-02
36	Fructose and mannose metabolism			5.91E-02
37	Isoflavonoid biosynthesis			2.31E-02
38	mRNA surveillance pathway			7.26E-02
39	Glutathione metabolism			6.01E-02

ComparisonA (25/13 ℃ vs. 13/13 ℃); ComparisonB (13/13℃ vs. 25/25 ℃); ComparisonC (25/13 ℃ vs. 25/25 ℃); DEGs ($\log_2^{Fold(change)}$ ≥ 1 or FDR ≤ 0.01).

Table S3. Mapman display of Significantly enriched DEGs in the DIF

Compa-rison	Bin	Name	DEGs number	up	down	p-value
A	28.1.3	DNA.synthesis/chromatin structure.histone	32	39	1	1.36E-07
A	28.1.3.2	DNA.synthesis/chromatin structure.histone.core	30	37	1	4.10E-07
A	17	hormone metabolism	100	69	38	9.06E-06
A	28.1	DNA.synthesis/chromatin structure	84	65	27	1.53E-04
A	27.3	RNA.regulation of transcription	279	140	148	5.07E-04
A	28.1.3.2.1	DNA.synthesis/chromatin structure.histone.core.H2A	16	16	0	5.07E-04
A	27	RNA	342	164	188	1.34E-03
A	20.1	stress.biotic	38	27	15	2.44E-03
A	31	cell	96	55	46	8.97E-03
A	28	DNA	112	51	74	8.97E-03
A	27.3.32	RNA.regulation of transcription.WRKY domain transcription factor family	20	18	2	8.97E-03
A	28.1.3.2.3	DNA.synthesis/chromatin structure.histone.core.H3	8	8	0	1.85E-02
B	20.2.1	stress.abiotic.heat	46	33	3	2.22E-05
B	16	secondary metabolism	134	112	35	1.69E-04
B	20.2	stress.abiotic	86	71	15	1.69E-04
B	16.2	secondary metabolism.phenylpropanoids	31	29	4	6.48E-03
B	28.1.3	DNA.synthesis/chromatin structure.histone	21	2	23	3.04E-02
B	16.2.1	secondary metabolism.phenylpropanoids. lignin biosynthesis	26	23	3	3.65E-02
B	28.1.3.2.1	DNA.synthesis/chromatin structure.histone.core.H2A	12	0	12	3.65E-02
B	28.1.3.2	DNA.synthesis/chromatin structure.histone.core	19	2	21	3.69E-02
B	16.8.2	secondary metabolism.flavonoids.chalcones	11	11	0	3.69E-02
B	16.1.5	secondary metabolism.isoprenoids.terpenoids	11	11	0	4.37E-02
C	16	secondary metabolism	117	101	33	3.19E-06
C	27.3.32	RNA.regulation of transcription.WRKY domain transcription factor family	22	21	1	3.51E-06
C	20	stress	128	102	34	1.22E-05
C	17	hormone metabolism	138	110	39	2.96E-05
C	20.2	stress.abiotic	77	60	17	2.56E-04
C	27.3	RNA.regulation of transcription	338	208	140	5.33E-04
C	16.2	secondary metabolism.phenylpropanoids	33	31	3	5.17E-03
C	20.2.1	stress.abiotic.heat	40	33	7	5.26E-03
C	26	misc	265	177	95	6.60E-03
C	17.6.1	hormone metabolism.gibberelin.synthesis- degradation	12	12	1	7.41E-03
C	11.8	lipid metabolism.'exotics'(steroids, squalene etc)	19	18	1	9.57E-03
C	27	RNA	397	233	174	1.00E-02
C	16.1.5	secondary metabolism.isoprenoids.terpenoids	8	8	0	1.01E-02
C	27.3.64	RNA.regulation of transcription.PHOR1	9	9	0	1.01E-02
C	17.6	hormone metabolism.gibberelin	19	19	3	1.29E-02
C	16.2.1	secondary metabolism.phenylpropanoids.lignin biosynthesis	27	24	3	3.92E-02
C	27.3.11	RNA.regulation of transcription.C2H2 zinc finger family	22	18	4	3.92E-02
C	11.8.1	lipid metabolism.'exotics' (steroids, squalene etc).sphingolipids	12	11	1	3.94E-02
C	34	transport	190	127	68	4.07E-02

Benjamini Hochberg corrected p-value less than 0.05. Up:number of up-regulated genes; Down: number of down-regulated genes. Comparison A (25/13℃ vs. 13/13 ℃); Comparison B (13/13℃ vs. 25/25 ℃); Comparison C (25/13℃ vs. 25/25℃) .

Table S4. sucrose synthesis and metabolism related genes of *D. officinale* PLBs under the DIF

Bincode	gene ID	Annotation	gene name	best hit Arabidopsis gene ID	A	B	C
2.2.1.5	dendrobium_glean_10105017	sucrose synthase4	*SUS4*	at3g43190	3.08	—	—
2.2.1.3.1	dendrobium_glean_10122358	Plant neutral invertase family protein	*A/N-INVD*	at1g22650	-2.47	1.72	—
34.2.1	dendrobium_glean_10087458	sucrose transporter 2	*SUT2*	at2g02860	—	1.55	1.70
34.2.1	dendrobium_glean_10083454	sucrose transporter 2	*SUT2*	at2g02860	—	-1.86	-1.89
34.2.1	dendrobium_glean_10033585	sucrose transporter 4	*SUT4*	at1g09960	—	-1.62	-1.39
34.2.1	dendrobium_glean_10009822	sucrose transporter 2	*SUT2*	at2g02860	—	2.78	—
2.2.1.4	dendrobium_glean_10127812	hexokinase 1	*HXK1*	at4g29130	—	-1.85	—
34.2.1	pequ_07166-d3	sucrose transporter 2	*SUT2*	at2g02860	—	—	-3.12
2.2.1.5	dendrobium_glean_10032668	sucrose synthase 4	*SUS4*	at3g43190	—	—	-1.04
2.2.1.4	dendrobium_glean_10013068	hexokinase-like 1	*HKL1*	at1g50460	—	—	-1.57

A:log_2FoldChange(25_13℃/13_13℃); B:log_2FoldChange(13_13℃/25_25℃); C:log_2FoldChange(25_13℃/25_25℃)

Table S5. The expression of DGEs on starch synthesis and metabolism of *D.officinale* PLBs under the DIF

Bincode	gene ID	Annotation	gene name	best hit Arabidopsis gene ID	A	B	C
2.2.2.2	dendrobium_glean_10135678	cytosolic alpha-glucan phosphorylase	*PHS2*	at3g46970	1.16	—	—
2.2.2.1	dendrobium_glean_10117747	heteroglycan glucosidase 1	*HGL1*	at3g23640	1.09	—	—
2.2.2.1	dendrobium_glean_10117749	heteroglycan glucosidase 1	*HGL1*	at3g23640	-1.65	—	-2.19
2.2.2.1.1	dendrobium_glean_10026401	alpha-amylase-like	*AMY1*	at4g25000	-1.28	—	—
2.2.2.3	dendrobium_glean_10143284	chloroplastidic phosphoglucan, water dikinase	*PWD*	at5g26570	-1.68	1.04	—
2.2.2.4	dendrobium_glean_10010642	Carbohydrate-binding-like fold		at5g01260	-1.13	1.52	—
2.2.2.6	dendrobium_glean_10108848	maltose transporter that is expressed in leaves and roots	*MEX1, RCP1*	at5g17520	-1.72	1.73	—
2.2.2.6	dendrobium_glean_10052668	maltose transporter that is expressed in leaves and roots	*MEX1, RCP1*	at5g17520	-1.38	1.54	—
2.1.2.1	dendrobium_glean_10082158	Glucose-1-phosphate adenylyltransferase large subunit	*APL2*	at1g27680	—	1.70	1.41
2.1.2.2	dendrobium_glean_10116809	starch synthase 4	*SS4*	at4g18240	—	1.54	—
2.2.2.1.1	dendrobium_glean_10054200	alpha-amylase-like 2	*AMY2*	at1g76130	—	-1.07	—
2.2.2.1.2	dendrobium_glean_10069668	beta-amylase 8	*BMY8,BAM3*	at4g17090	—	2.22	1.41
2.2.2.1.2	dendrobium_glean_10050481	beta-amylase 1	*BAM1,BMY7*	at3g23920	—	2.77	2.91
2.2.2.1.2	dendrobium_glean_10015532	beta-amylase 1	*BAM1,BMY7*	at3g23920	—	3.00	3.13
2.1.2.2	dendrobium_glean_10016137	starch synthase 3	*SS3*	at1g11720	—		1.24

A:log_2FoldChange(25_13℃/13_13℃); B:log_2FoldChange(13_13℃/25_25℃); C:log_2FoldChange(25_13℃/25_25℃)

Table S6. Expression of DEGs in flavonols medabolism of *Dendrobium officinale* PLBs under the DIF

Bin	gene id	description	gene name	best hit Arabidopsis gene ID	A	B	C
16.8.4	dendrobium_glean_10022776	Don-glucosyltransferase1,UDP-glucosyltransferase 73C5	*DOGT1,UGT73C5*	at2g36800	—	—	1.35
16.8.4	dendrobium_glean_10108071	Don-glucosyltransferase1,UDP-glucosyltransferase 73C5	*DOGT1,UGT73C5*	at2g36800	2.17	-2.80	—
16.8.4.1	dendrobium_glean_10067667	flavonol synthase 1	*FLS1*	at5g08640	-3.54	5.30	1.76
16.8.4.1	dendrobium_glean_10071537	flavonol synthase 1	*FLS1*	at5g08640	-3.81	5.56	1.75
16.8.4	dendrobium_glean_10021516	2-oxoglutarate (2OG) and Fe(II)-dependent oxygenase superfamily protein, gain-of-function in ABA-modulated seed germination 2, germination insensitive to ABA mutant 2	*GAS2,GIM2*	at2g36690	—	—	-1.18
16.8.4	dendrobium_glean_10060927	2-oxoglutarate (2OG) and Fe(II)-dependent oxygenase superfamily protein, gain-of-function in ABA-modulated seed germination 2, germination insensitive to ABA mutant 2	*GAS2,GIM2*	at2g36690	2.49	—	—
16.8.4	dendrobium_glean_10124444	2-oxoglutarate (2OG) and Fe(II)-dependent oxygenase superfamily protein, gain-of-function in ABA-modulated seed germination 2, germination insensitive to ABA mutant 2	*GAS2,GIM2*	at2g36690	—	—	-1.21
16.8.4	dendrobium_glean_10124445	2-oxoglutarate (2OG) and Fe(II)-dependent oxygenase superfamily protein, Ggain-of-function in ABA-modulated seed germination 2, germination insensitive to ABA mutant 2	*GAS2,GIM2*	at2g36690	2.28	-1.79	—
16.8.4	dendrobium_glean_10026429	senescence-related gene 1	*SRG1*	at1g17020	—	1.78	2.59
16.8.4.2	dendrobium_glean_10052239	UDP-glucosyl transferase 73B1	*UGT73B1*	at4g34138	—	-1.07	—
16.8.4.2	dendrobium_glean_10052234	UDP-glucosyl transferase 73B3	*UGT73B3*	at4g34131	—	—	-1.01
16.8.4.2	dendrobium_glean_10132500	UDP-glucosyl transferase 73B3	*UGT73B3*	at4g34131	—	—	-1.52
16.8.4.2	dendrobium_glean_10025842	UDP-glycosyltransferase 73B4	*UGT73B4*	at2g15490	—	1.50	2.46
16.8.4.2	dendrobium_glean_10052238	UDP-glycosyltransferase 73B4	*UGT73B4*	at2g15490	—	-1.14	—
16.8.4.2	dendrobium_glean_10052240	UDP-glycosyltransferase 73B4	*UGT73B4*	at2g15490	—	-1.88	-1.77
16.8.4.2	dendrobium_glean_10070561	UDP-glycosyltransferase 73B4	*UGT73B4*	at2g15490	—	-2.07	-2.06
16.8.4.2	dendrobium_glean_10132486	UDP-glycosyltransferase 73B4	*UGT73B4*	at2g15490	—	5.39	—
16.8.4	dendrobium_glean_10132494	UDP-glycosyltransferase 73B4	*UGT73B4*	at2g15490	1.54	—	—
16.8.4.2	dendrobium_glean_10132494	UDP-glycosyltransferase 73B4	*UGT73B4*	at2g15490	1.54	—	—
16.8.4	dendrobium_glean_10055223	UDP-glucosyl transferase 73B5	*UGT73B5*	at2g15480	—	-1.98	-1.06
16.8.4.2	dendrobium_glean_10055223	UDP-glucosyl transferase 73B5	*UGT73B5*	at2g15480	—	-1.98	-1.06
16.8.4	dendrobium_glean_10132499	UDP-glucosyl transferase 73B5	*UGT73B5*	at2g15480	-1.20	—	-1.98
16.8.4.2	dendrobium_glean_10132499	UDP-glucosyl transferase 73B5	*UGT73B5*	at2g15480	-1.20	—	-1.98
16.8.4	dendrobium_glean_10067948	UDP-glucosyl transferase 73C1	*UGT73C1*	at2g36750	1.24	—	—
16.8.4	dendrobium_glean_10079736	UDP-glucosyl transferase 73C6	*UGT73C6*	at2g36790	—	—	1.46

16.8.4	dendrobium_glean_10081749	UDP-glucosyl transferase 73C6	*UGT73C6*	at2g36790	2.54	-1.98	—
16.8.4	dendrobium_glean_10081750	UDP-glucosyl transferase 73C6	*UGT73C6*	at2g36790	1.48	—	1.08
16.8.4	dendrobium_glean_10084964	UDP-glucosyl transferase 73C6	*UGT73C6*	at2g36790	—	2.11	1.98
16.8.4	dendrobium_glean_10084962	UDP-glucosyl transferase 73D1	*UGT73D1*	at3g53150	2.23	—	2.36
16.8.4	dendrobium_glean_10084968	UDP-glucosyl transferase 73D1	*UGT73D1*	at3g53150	2.06	—	1.31
16.8.4.3	dendrobium_glean_10064134	UDP-glucosyl transferase 78D2	*UGT78D2*	at5g17050	-1.81	2.53	—
16.8.4.2	dendrobium_glean_10093854	UDP-glucosyl transferase 88A1	*UGT88A1*	at3g16520	—	1.54	1.25
16.8.4	dendrobium_glean_10008391	2-oxoglutarate (2OG) and Fe(II)-dependent oxygenase superfamily protein		at1g78550	1.72	—	2.59
16.8.4	dendrobium_glean_10108072	UDP-glycosyltransferase 73C6-like	*UGT73C6*	at2g36780	—	-3.51	-9.13
16.8.4.2	dendrobium_glean_10108072	UDP-glycosyltransferase 73C6-like	*UGT73C6*	at2g36780	—	-3.51	-9.13
16.8.4	dendrobium_glean_10124455	2-oxoglutarate (2OG) and Fe(II)-dependent oxygenase superfamily protein		at2g44800	—	-1.06	—
16.8.4	dendrobium_glean_10033155	2-oxoglutarate (2OG) and Fe(II)-dependent oxygenase superfamily protein		at4g25300	—	2.14	2.70
16.8.4	dendrobium_glean_10047039	2-oxoglutarate (2OG) and Fe(II)-dependent oxygenase superfamily protein		at4g25310	1.49	—	2.19
16.8.4	dendrobium_glean_10080291	flavanone 3 hydroxylase	*F3H*	at5g20400	—	—	1.11

A:log_2FoldChange(25_13℃/13_13℃); B:log_2FoldChange(13_13℃/25_25℃); C:log_2FoldChange(25_13℃/25_25℃)

Table S7 Expression of DEGs in Heat stress of *D. officinale* PLBs

BinID	BinNumber	Annotation	gene name	best hit Arabidopsis gene ID	A	B	C
20.2.1	dendrobium_glean_10092409	DNAJ heat shock N-terminal domain-containing protein, DNA J protein C76	*ATDJC17,DJC76*	at5g23240	-1.26	2.70	1.44
20.2.1	dendrobium_glean_10092411	DNAJ heat shock N-terminal domain-containing protein, DNA J protein C76	*ATDJC17,DJC76*	at5g23240	-1.26	2.39	—
20.2.1	dendrobium_glean_10030679	DNAJ heat shock N-terminal domain-containing protein, thermosensitive male sterile 1	*ATERDJ3A,TMS1*	at3g08970	—	1.53	—
20.2.1	dendrobium_glean_10020260	DNAJ heat shock N-terminal domain-containing protein, thermosensitive male sterile 1	*ATERDJ3A,TMS1*	at3g08970	—	1.45	—
20.2.1	dendrobium_glean_10042812	J-domain protein 6	*ATJ6*	at5g06910	—	1.58	1.65
20.2.1	dendrobium_glean_10107202	luminal binding protein, ER-localized member of the HSP70 family	*BIP1*	at5g28540	1.08	2.05	—
20.2.1	dendrobium_glean_10072107	luminal binding protein, ER-localized member of the HSP70 family	*BIP1*	at5g28540	-1.58	—	—
20.2.1	dendrobium_glean_10072106	Luminal binding protein	*BIP2*	at5g42020	—	2.35	1.36
20.2.1	dendrobium_glean_10030985	Luminal binding protein	*BIP2*	at5g42020	—	1.64	1.23
20.2.1	dendrobium_glean_10059032	casein lytic proteinase B3, Hsp101 homologue, albino and pale green 6	*CLPB3,APG6,CLPB-P*	at5g15450	-1.77	2.21	—
20.2.1	dendrobium_glean_10031865	casein lytic proteinase B3, Hsp101 homologue, albino and pale green 6	*CLPB3,APG6,CLPB-P*	at5g15450	-3.67	2.06	-1.61
20.2.1	dendrobium_glean_10031800	casein lytic proteinase B4, Clp/Hsp100, casein lytic proteinase B-M	*CLPB4,CLPB-M,HSP98.7*	at2g25140	1.73	—	1.40
20.2.1	dendrobium_glean_10096759	DNAJ heat shock family protein, DNA J protein A6	*DJA6*	at2g22360	-1.37	—	—
20.2.1	dendrobium_glean_10026169	Chaperone DnaJ-domain superfamily protein	*DJC53*	at1g56300	-1.17	1.90	—
20.2.1	dendrobium_glean_10070818	Chaperone DnaJ-domain superfamily protein	*DJC53*	at1g56300	—	1.13	1.75
20.2.1	dendrobium_glean_10082564	Chaperone DnaJ-domain superfamily protein	*DJC73*	at5g59610	—	1.22	—
20.2.1	dendrobium_glean_10004216	Chaperone DnaJ-domain superfamily protein	*DJC73*	at5g59610	—	1.08	—
20.2.1	dendrobium_glean_10083783	DNAJ heat shock family protein,DNAJ PROTEIN	*DNAJ*	at2g20560	-1.52	1.89	—
20.2.1	dendrobium_glean_10095728	DNAJ heat shock family protein	*DNAJ*	at2g20560	—	—	-1.47
20.2.1	dendrobium_glean_10019860	DNAJ heat shock family protein	*DNAJ*	at2g20560	—	—	1.13
20.2.1	dendrobium_glean_10110883	heat shock protein 70 (Hsp 70) family protein, early-responsive to dehydration2, heat shock protein 70T-1	*ERD2,HSP70T-1*	at1g56410	-1.37	—	—
20.2.1	dendrobium_glean_10093778	heat shock protein 90.2, heat shock protein 81.2	*ERD8,HSP81.2,HSP90.2*	at5g56030	-1.06	—	—
20.2.1	dendrobium_glean_10095987	DNAJ heat shock N-terminal domain-containing protein, gravitropism defetive 2, katamari 2	*GRV2,KAM2,SGR8*	at2g26890	—	-6.27	-6.27

20.2.1	dendrobium_glean_10035693	gravitropism defective 2, katamari 2	*GRV2,KAM2,SGR8*	at2g26890	—	7.27	—
20.2.1	dendrobium_glean_10115433	heat shock cognate protein 70-1	*HSC70-1,HSP70-1*	at5g02500	—	2.27	2.39
20.2.1	dendrobium_glean_10092271	heat shock factor 1	*HSF1,HSFA1A*	at4g17750	2.99	—	8.55
20.2.1	dendrobium_glean_10111029	heat shock factor 1,class A heat shock factor 1A	*HSF1,HSFA1A*	at4g17750	—	2.16	1.37
20.2.1	dendrobium_glean_10092269	heat shock factor 3,class A heat shock factor 1 1B	*HSF3,HSFA1B*	AT5G16820	1.37	—	1.52
20.2.1	dendrobium_glean_10031759	heat shock factor 4,class A heat shock factor B1	*HSF4,HSFB1*	at4g36990	—	3.50	3.50
20.2.1	dendrobium_glean_10059581	winged-helix DNA-binding transcription factor family protein, heat shock transcrition factor B2B	*HSF7, HSFB2B*	at4g11660	-1.21	1.28	—
20.2.1	dendrobium_glean_10086923	heat shock transcription factor A2	*HSFA2*	at2g26150	-1.23	1.18	—
20.2.1	dendrobium_glean_10039865	heat shock transcription factor A2	*HSFA2*	at2g26150	1.25	—	1.48
20.2.1	dendrobium_glean_10012441	heat shock protein 101	*HSP101,HOT1,CLpB1*	at1g74310	—	—	1.59
20.2.1	dendrobium_glean_10071770	heat shock protein 101	*HSP101,HOT1,CLpB1*	at1g74310	—	1.20	—
20.2.1	dendrobium_glean_10084732	heat shock protein 17.4	*HSP17.4*	at3g46230	-2.97	3.46	—
20.2.1	dendrobium_glean_10096238	HSP20-like chaperones superfamily protein	*HSP17.4B*	at1g54050	—	2.65	2.39
20.2.1	dendrobium_glean_10074943	17.6 kDa class II heat shock protein	*HSP17.6II*	at5g12020	-2.22	2.28	—
20.2.1	dendrobium_glean_10019288	17.6 kDa class II heat shock protein	*HSP17.6II*	at5g12020	-1.19	2.33	1.14
20.2.1	dendrobium_glean_10042127	heat shock protein 17.6A	*HSP17.6A*	at5g12030	-1.71	3.19	1.48
20.2.1	dendrobium_glean_10042128	heat shock protein 17.6A	*HSP17.6A*	at5g12030	-1.78	3.54	1.76
20.2.1	dendrobium_glean_10024324	HSP20-like chaperones superfamily protein	*HSP17.6B*	at2g29500	—	—	-1.42
20.2.1	dendrobium_glean_10056722	HSP20-like chaperones superfamily protein	*HSP17.6B*	at2g29500	—	4.90	—
20.2.1	dendrobium_glean_10087466	HSP20-like chaperones superfamily protein	*HSP17.6C*	at1g53540	—	1.03	—
20.2.1	dendrobium_glean_10087467	HSP20-like chaperones superfamily protein	*HSP17.6C*	at1g53540	—	1.19	—
20.2.1	dendrobium_glean_10042129	17.6 kDa class II heat shock protein	*HSP17.6II*	at5g12020	—	3.40	2.89
20.2.1	dendrobium_glean_10087095	heat shock protein 18.2	*HSP18.2*	at5g59720	9.29	—	4.38
20.2.1	dendrobium_glean_10087099	heat shock protein 18.2	*HSP18.2*	at5g59720	-1.34	2.40	1.06
20.2.1	dendrobium_glean_10016786	heat shock protein 18.2	*HSP18.2*	at5g59720	-2.85	3.43	—

20.2.1	dendrobium_glean_10024322	HEAT SHOCK PROTEIN 18.2	*HSP18.2*	at5g59720	—	1.78	1.79
20.2.1	dendrobium_glean_10000820	HEAT SHOCK PROTEIN 18.2	*HSP18.2*	at5g59720	—	2.39	2.77
20.2.1	dendrobium_glean_10056824	heat shock protein 21	*HSP21*	at4g27670	-3.45	3.55	—
20.2.1	dendrobium_glean_10032180	heat shock protein 21	*HSP21*	at4g27670	-1.20	1.80	—
20.2.1	dendrobium_glean_10087465	HSP20-like chaperones superfamily protein	*HSP22.0*	at4g10250	-3.22	3.48	—
20.2.1	dendrobium_glean_10072261	heat shock protein 70	*HSP70*	at3g12580	—	3.05	2.55
20.2.1	dendrobium_glean_10032708	heat shock protein 70	*HSP70*	at3g12580	—	1.11	1.99
20.2.1	dendrobium_glean_10032713	heat shock protein 70	*HSP70*	at3g12580	—	2.85	2.12
20.2.1	dendrobium_glean_10066677	heat shock cognate protein 70	*HSP70*	at5g02500	1.12	-1.12	—
20.2.1	dendrobium_glean_10045076	heat shock cognate protein 70	*HSP70*	at5g02500	1.89	—	2.80
20.2.1	dendrobium_glean_10130811	DNAJ homologue 3	*J3*	at3g44110	-1.60	—	—
20.2.1	dendrobium_glean_10023471	DNAJ homologue 3	*J3*	at3g44110	-1.99	—	-1.11
20.2.1	dendrobium_glean_10074235	DNAJ homologue 3	*J3*	at3g44110	—	2.55	2.76
20.2.1	dendrobium_glean_10125727	Chaperone DnaJ-domain superfamily protein,DNA J PROTEIN C22,TRANSLOCON AT THE OUTER ENVELOPE MEMBRANE OF CHLOROPLASTS 12	*J8,DJC22,TOC12,*	at1g80920	1.03	—	1.08
20.2.1	dendrobium_glean_10001340	Chaperone protein htpG family protein，encodes an ortholog of GRP94, an ER-resident HSP90-like protein,ATHSP90.7	*SHD*	at4g2419	1.36	—	1.27
20.2.1	dendrobium_glean_10012972	Double Clp-N motif-containing P-loop nucleoside triphosphate hydrolases superfamily protein,SUPPRESSOR OF MAX2 1	*SMAX1*	at5g57710	1.69	—	1.58
20.2.1	dendrobium_glean_10112299	Double Clp-N motif-containing P-loop nucleoside triphosphate hydrolases superfamily protein,SMAX1-LIKE 7	*SMXL7*	at2g29970	1.42	-1.36	—
20.2.1	dendrobium_glean_10131412	DNAJ heat shock N-terminal domain-containing protein		at1g21080	1.24	—	—
20.2.1	dendrobium_glean_10109992	Heat shock protein 70 (Hsp 70) family protein		at3g09440	—	2.22	3.01
20.2.1	dendrobium_glean_10108063	heat shock protein		at3g22530	—	—	-1.16
20.2.1	dendrobium_glean_10087134	HSP20-like chaperones superfamily protein		at4g21870	-1.62	—	—
20.2.1	dendrobium_glean_10065596	DNAJ heat shock family protein		at5g25530	-4.08	—	-2.88

A:log_2FoldChange(25_13℃/13_13℃); B:log_2FoldChange(13_13℃/25_25℃); C:log_2FoldChange(25_13℃/25_25℃)
