# Supplementary figures and images for: Transcriptomic Analysis for Diurnal Temperature Differences Reveals Gene-Regulation-Network Response to Accumulation of Bioactive Ingredients of Protocorm-like Bodies in Dendrobium officinale
Source: Plants (Basel). 2024 Mar 18;13(6):874. doi: 10.3390/plants13060874 (PMC10975105; doi:10.3390/plants13060874)

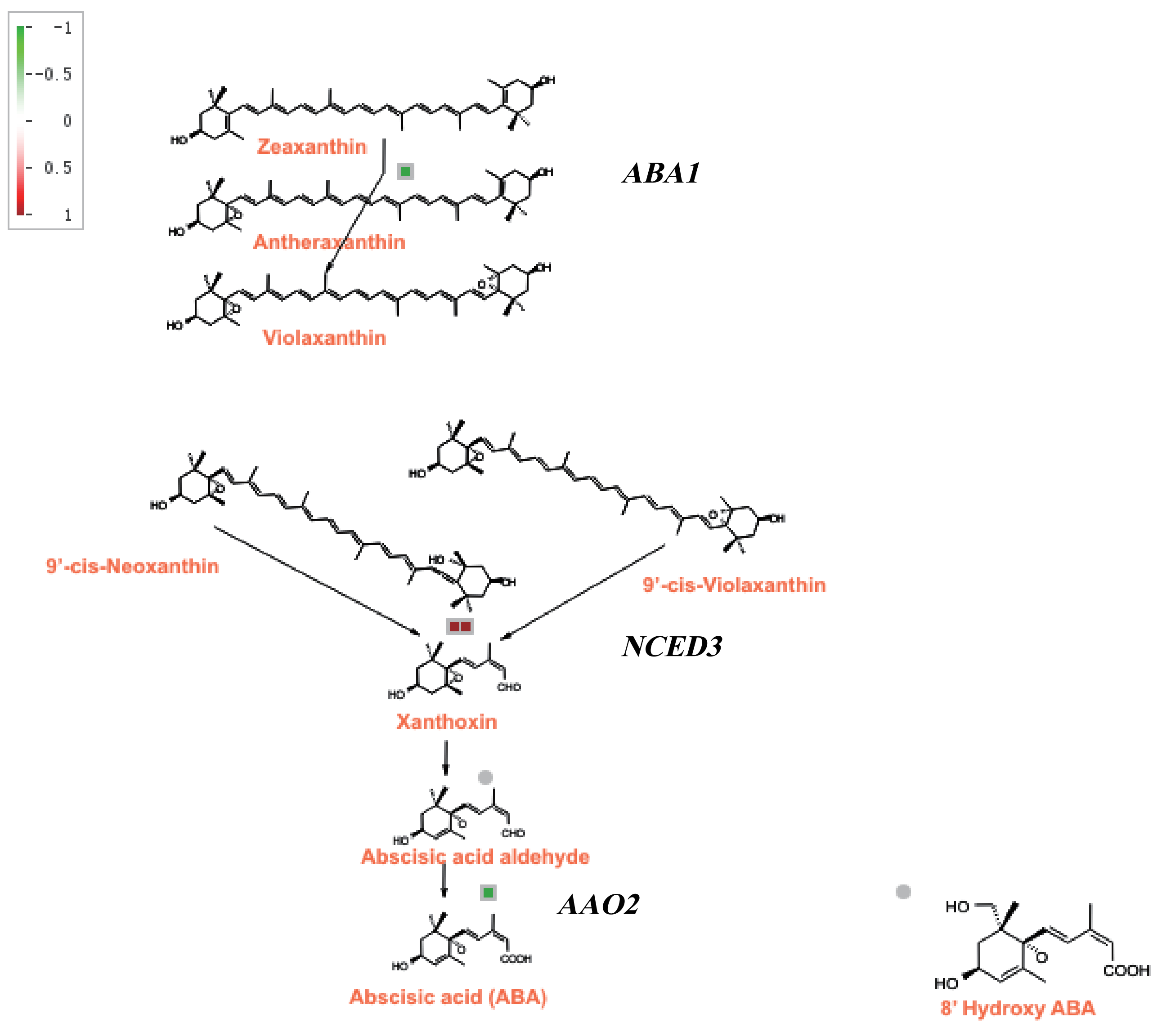

Supplement: Supplementary file 1 [file plants-13-00874-s001.zip › Figure S1.png]

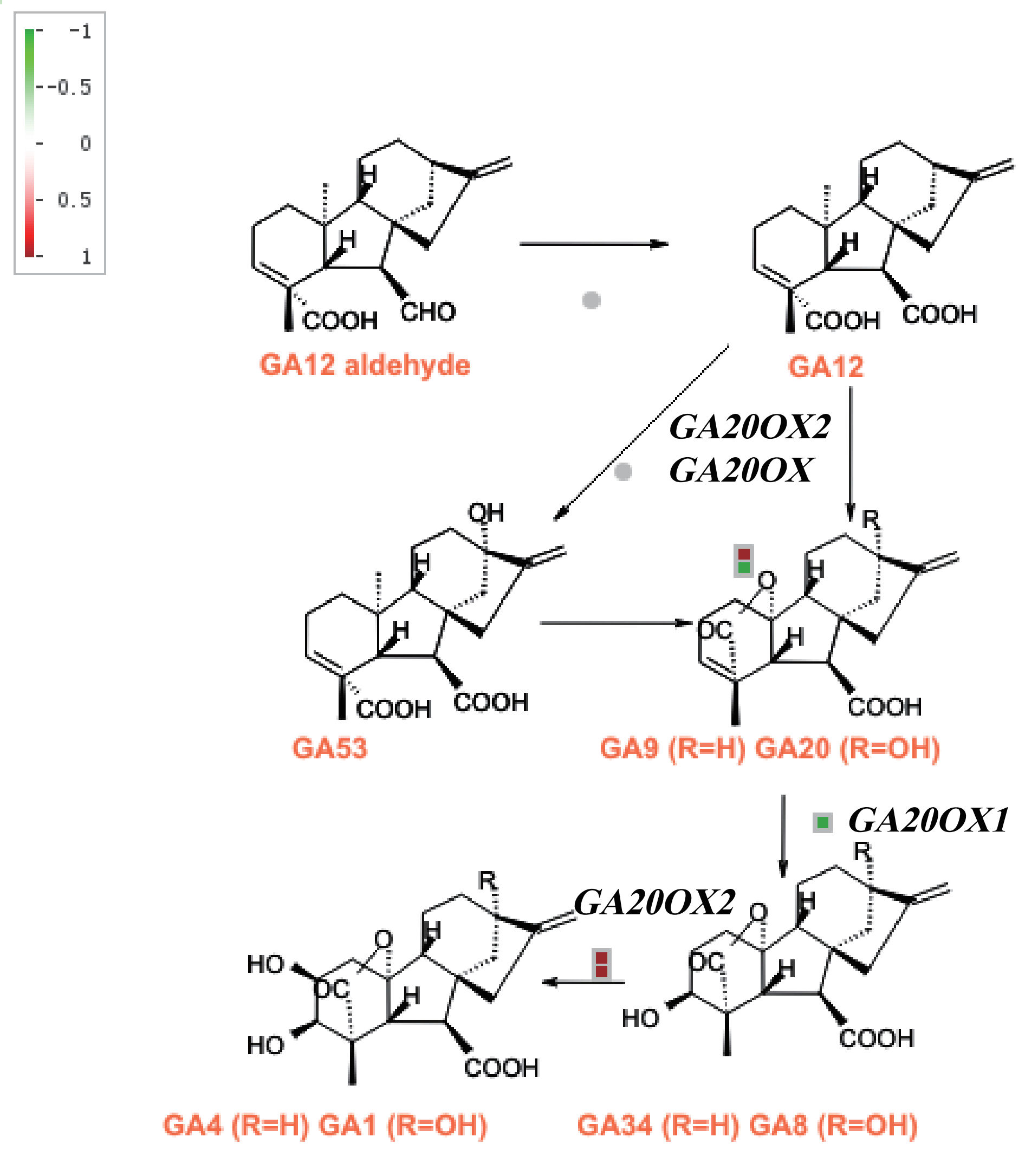

Supplement: Supplementary file 1 [file plants-13-00874-s001.zip › Figure S2.png]

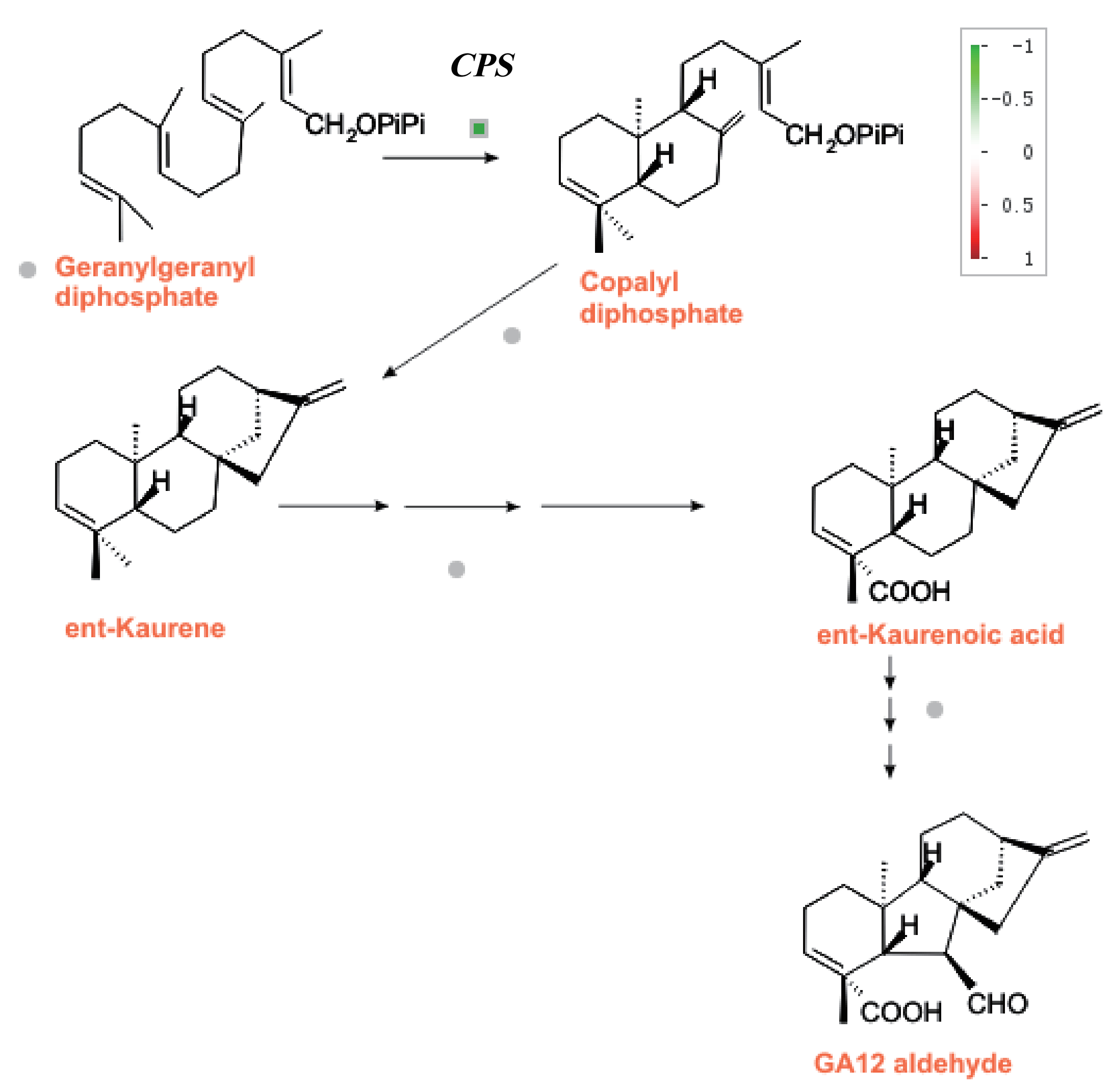

Supplement: Supplementary file 1 [file plants-13-00874-s001.zip › Figure S3.png]

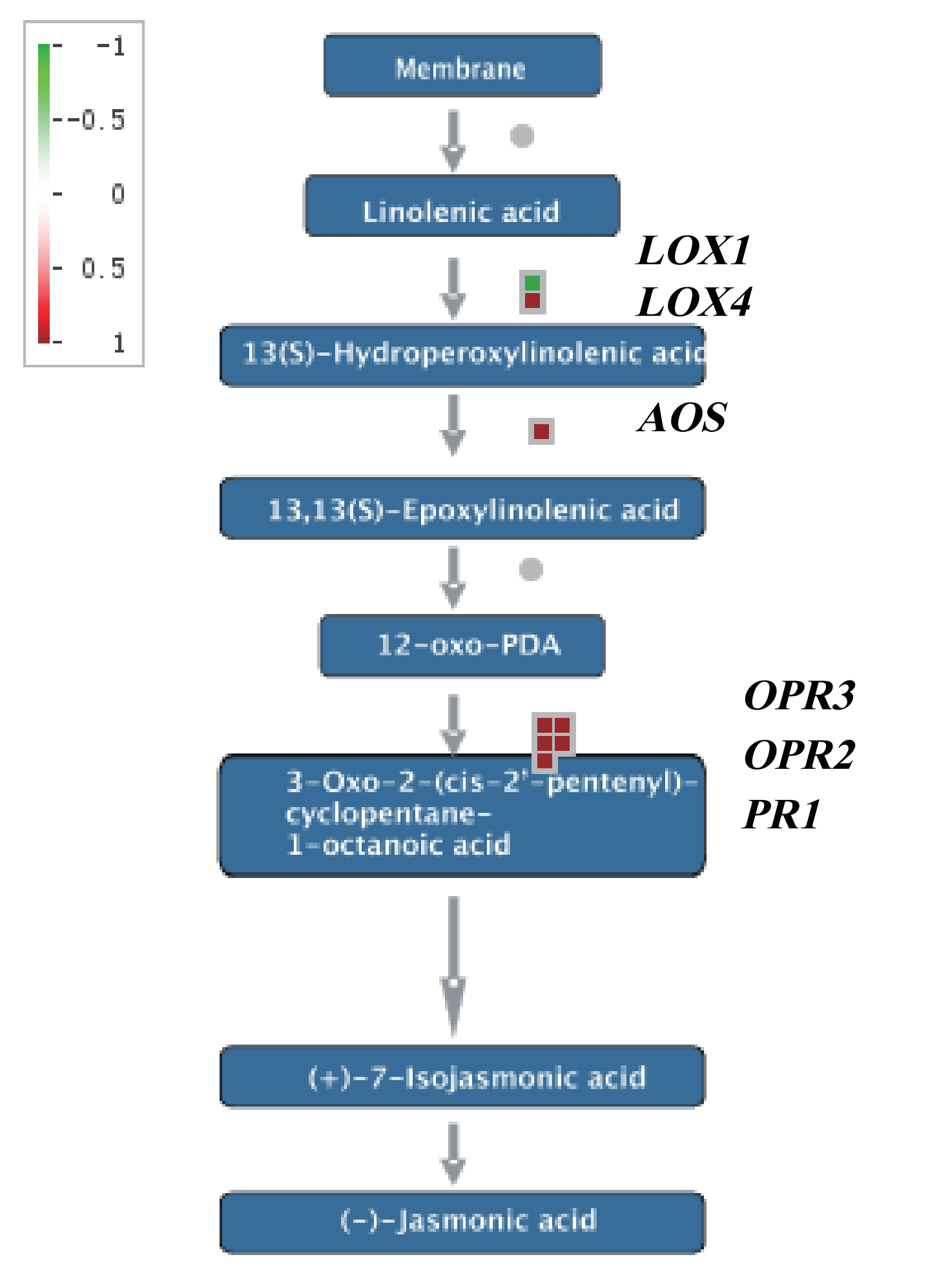

Supplement: Supplementary file 1 [file plants-13-00874-s001.zip › Figure S4.png]
